# Clinical analysis of incomplete rupture of the uterus secondary to previous cesarean section

**DOI:** 10.1515/med-2024-0927

**Published:** 2024-04-01

**Authors:** Hong Yang, Yun Zhao, Jiahui Tu, Yanan Chang, Chanyun Xiao

**Affiliations:** Department of Obstetrics, Maternal and Child Health Hospital of Hubei Province, Wuhan City, Hubei Province, 430070, P. R. China; Women’s Health Unit, Maternal and Child Health Hospital of Hubei Province, Wuhan City, Hubei Province, 430070, P. R. China; Department of Obstetrics, Maternal and Child Health Hospital of Hubei Province, No. 745 Wuluo Road, Hongshan District, Wuhan City, Hubei Province, 430070, P. R. China

**Keywords:** cesarean section, uterine rupture, myometrium thickness, ultrasound

## Abstract

Uterine rupture is a rupture of the body or lower part of the uterus during pregnancy or delivery. Total of 98 cases with incomplete uterine rupture were classified as the incomplete uterine rupture group, 100 cases with a history of cesarean delivery without uterine rupture were classified as the non-ruptured uterus group, and controls were selected using a systematic sampling method. The maternal age ≥35 years were associated with 2.18 times higher odds of having an incomplete uterine rupture. The odd of having an incomplete uterine rupture was 3.744 times higher for a woman with delivery interval ≤36 months. Having pregnancy complication was associated with 3.961 times higher odds of having an incomplete uterine rupture. The neonatal weight was lighter in the incomplete uterine rupture group (*P* = 0.007). The number of preterm birth and transfer to the NICU were higher in the incomplete uterine rupture group (*P* ＜ 0.01). The operation time and the length of time in hospital were longer in the group with incomplete uterine rupture (*P* ＜ 0.01). Age ≥35 years, delivery interval ≤36 month, and pregnancy with complication were independent risk factors of incomplete rupture of the uterus secondary to previous cesarean section.

## 1 Introduction

Uterine rupture is a catastrophic obstetric emergency that can lead to maternal and neonatal death in severe cases [1]. The incidence of uterine rupture varies widely in the published literature because of the study population base and the definition given. According to WHO (World Health Organization), the average incidence of uterine rupture is 5.3/10,000. Globally, the incidence of uterine rupture is 0.07% with the tendency of being lower in the developed countries than the developing countries [2]. The incidence was 0.18% in 96 hospitals covering most regions of China in 2015–2016 [3]. The most common risk factor is scarred uterus, usually due to a previous cesarean section, with rupture occurring mostly in the anterior lower uterine segment [4].

According to the analysis of national maternal and child health care statistics, the cesarean section rate in China rose from 28.8% in 2008 to 34.9% in 2014 and reached 36.7% in 2018 [5], even in some megalopolis the cesarean rate is greater than 60% [6]. Selective repeat cesarean section has emerged as a major cause of high cesarean section incidence in many countries [7]. In general, complete uterine rupture was defined as a laceration in all layers of the uterine wall, including the plasma membrane and amniotic membrane. Incomplete uterine rupture was defined as a tear in the layer of the muscle but the plasma membrane or amniotic membrane remained intact [8,9].

In this study, we found that there was a gradual increase in the number of incomplete uterine ruptures secondary to a history of previous cesarean delivery, such cases were collected in our hospital in the past 4 years for study, with the aim of early detection and prevention of such incomplete uterine ruptures.

## 2 Materials and methods

### 2.1 Study setting

Total 98 cases of incomplete uterine rupture recorded in Hubei Provincial Maternal and Child Health Hospital, Wuhan, China from 2018 to 2021 were collected and retrospectively analyzed, and 100 cases with a history of cesarean delivery during the same period were selected to elective repeat cesarean delivery without uterine rupture as the non-ruptured uterus group. This hospital is one of the critical maternity transfer centers in Hubei Province, with an average of more than 20,000 deliveries per year in recent years. All aspects of this study were conducted under the approval of Maternal and Child Health Hospital of Hubei Province.

### 2.2 Inclusion criteria

Delivery records of all pregnant women with a history of at least one previous cesarean section and diagnosed with incomplete uterine rupture during the current cesarean section were retrieved and included in the study as the incomplete uterine rupture group. A systematic sampling method was used to select cases of scarred uterus with previous cesarean delivery who underwent cesarean delivery without uterine rupture at our hospital during the same period in a ratio of 1:1 to be included in the control group. All cases had a complete medical history and auxiliary examination findings.

### 2.3 Variables of the study

- Socio-demographic variables: age, height, weight, and BMI.
- Obstetric factors: gravida, para, and gestational age, interval time since previous cesarean section, pregnancy-related complications such as hypertension (including pre-eclampsia), diabetes, history of hysteroscopic surgery (such as endometrial polyposectomy, intrauterine adhesiolysis, subserosal myomectomy), duration of surgery, bleeding, number of days in hospital, postoperative infection.
- Neonatal outcome: preterm birth, weight, gestational week, transfer to NICU.
- Auxiliary examination: ultrasound measurement of the thickness of the lower myometrium by abdominal ultrasound within 24 h before hospitalization and delivery.

### 2.4 Statistical analysis

Quantitative data were expressed in mean ± SD. Statistical analyses were performed using SPSS for 23.0. Quantitative data were compared using student’s *t*-test for continuous variables and chi-square tests for categorical variables. Multivariable logistic regression was used to analyze the relationship between maternal conditions and uterine rupture. Receiver operating curve (ROC) analyses were used to determine optimal cut-off values for sensitivity and specificity. The significant difference was pre-set at *P* < 0.05.

## 3 Results

### 3.1 Characteristics of participants

This study revealed that age ≥35 years, multiparous, number of previous cesarean section ≥2, delivery interval ≤36 months, history of hysteroscopic surgery, women with pregnancy complications were highly proportionate among cases of incomplete uterine rupture compared to controls (*P* < 0.05).

The proportion of age ≥35 years was 40 (40.8%) among cases and 21 (21%) among controls. Incomplete uterine rupture cases of 32 (32.7%) and 13 (13%) of controls belonged to gravidity of three and above. Women with number of previous cesarean section ≥2 were slightly higher in proportion among cases of incomplete uterine rupture 14(14.3%) compared to controls 4(4%). Delivery interval ≤36 months was highly proportionate among cases with 43 (43.9%) whereas 18 (18%) among controls. The proportion of women with history of hysteroscopic surgery is slightly higher among cases 10 (10.2%) compared to controls 3 (3%). Women with pregnancy complication were higher in proportion among cases of incomplete uterine rupture 49(50%) compared to controls 21(21%) (Table 1).

**Table 1:** Comparison of demographic and obstetric characteristics of pregnant women with incomplete ruptured uterus secondary to previous cesarean section

Variables	Incomplete uterine ruptures group (*n* = 98)	Non-ruptured uterus group (*n* = 100)	*P* value
Maternal age (year)	33.74 ± 3.33	32.30 ± 3.15	**0.002**
Age ≥35 years			**0.003**
Yes	40 (40.8%)	21 (21%)	
No	58 (59.2%)	79 (79%)	
Pre-pregnancy BMI (kg/m^2^)	22.06 ± 2.63	22.99 ± 3.87	0.05
Gravidity >3			**0.005**
Yes	32 (32.7%)	13 (13%)	
No	66 (67.3%)	87 (87%)	
Number of previous cesarean section ≥2			**0.014**
Yes	14 (14.3%)	4 (4%)	
No	84 (85.7%)	96 (96%)	
Delivery interval			**<0.001**
≤36 months	43 (43.9%)	18 (18%)	
>36 months	55 (56.1%)	81 (81%)	
History of hysteroscopic surgery			**0.048**
Yes	10 (10.2%)	3 (3%)	
No	88 (89.8%)	97 (97%)	
Pregnancy complication			**<0.001**
Yes	49 (50%)	21 (21%)	
No	49 (50%)	79 (79%)	

BMI, body mass index. Calculating formula: BMI = Weight (kg) ÷ Height^2^ (m).

*P* values in bold indicate that the difference was significant.

### 3.2 Factors associated with incomplete ruptured uterus secondary to previous cesarean section

The odds of happening incomplete uterine rupture in relation to different characteristics of women were estimated by odds ratio using multivariate logistic regression analysis (Table 2). The result of multivariate analysis maternal age ≥35 years were associated with 2.18 (AOR = 2.18; 95% confidence interval [CI]: 1.059, 4.490) times higher odds of having an incomplete uterine rupture compared to maternal age <35 years. The odd of having an incomplete uterine rupture was 3.744 (AOR = 3.744; 95% CI: 1.828, 7.665) times higher for a woman with delivery interval ≤36 months. Having pregnancy complication was associated with 3.961 (AOR = 3.961; 95% CI: 1.989, 7.889).

**Table 2:** Multivariate logistic regression analysis for factors associated with incomplete ruptured uterus secondary to previous cesarean section

Factor	*B*	*P* value	Adjust odds ratio	95% CI lower	95% CI upper
Age ≥35 years	0.780	**0.034**	2.180	1.059	4.490
Parity >3	0.673	0.132	1.959	0.816	4.704
Number of previous cesarean section ≥2	0.791	0.233	2.205	0.601	8.084
Delivery interval ≤36 months	1.320	**0.000**	3.744	1.828	7.665
History of hysteroscopic surgery	0.304	0.683	1.356	0.315	5.834
Pregnancy complication	1.376	**0.000**	3.961	1.989	7.889

*P* values in bold indicate that the difference was significant (*P* < 0.05).

### 3.3 Maternal and neonatal outcomes

There were no maternal deaths and hysterectomy secondary to incomplete uterine rupture. Maternal and neonatal outcomes are shown in Table 3. The gestational weeks of incomplete uterine rupture group and non-ruptured uterus group were 37.29 ± 1.26 weeks and 38.50 ± 0.52 weeks, respectively (*P* ＜ 0.001). The neonatal weight was lighter in the incomplete uterine rupture group than in the control group (*P* = 0.007). The number of preterm birth was highly proportionate among cases with 31 (31.6%) whereas 1 (1%) among controls. The number of cases (11, 11.2%) transferred to the NICU was higher than controls (1, 1%). The main reasons for referral to the neonatal unit are neonatal asphyxia and neonatal respiratory distress. The operation time and the length of time in hospital were longer in the group with incomplete uterine rupture than in the control group (*P* ＜ 0.01). The number of placental adhesions in the incomplete uterine rupture group was 21 (21.4%) compared to 5 (5%) in the control group. The differences in intraoperative bleeding and the number of postoperative infections between the two groups were not statistically significant (*P* > 0.05). The difference in ultrasound measurement of myometrial thickness values of the lower uterine segment between the two groups was statistically significant (*P* ＜ 0.001).

**Table 3:** Comparison of maternal and neonatal outcomes

Variables	Incomplete uterine ruptures group (*n* = 98)	Non-ruptured uterus group (*n* = 100)	*P* value
Gestational weeks	37.29 ± 1.26	38.50 ± 0.52	<**0.001**
Neonatal weight (g)	3136.78 ± 420.26	3282.70 ± 334.235	**0.007**
Preterm birth			<**0.001**
	31 (31.6%)	1 (1%)	
	67 (68.4%	99 (99%)	
Transfer to the NICU			**0.003**
	11 (11.2%)	1 (1%)	
	87 (88.8%)	99 (99%)	
Operating time (min)	41.10 ± 9.83	37.68 ± 6.22	**0.002**
Hospitalization time (day)	6.91 ± 4.02	5.38 ± 0.88	<**0.001**
Placenta adhesion			**0.001**
Yes	21 (21.4%)	5 (5%)	
No	77 (78.6%)	95 (95%)	
Intraoperative blood Loss (mL)	325.00 ± 86.23	317.00 ± 46.17	0.415
Postoperative infection			0.366
Yes	3 (3.1%)	1 (1%)	
No	95 (96.9%)	99 (99%)	
Thickness of the lower uterine myometrium (mm)	0.84 ± 0.50	1.61 ± 0.51	<**0.001**

*P* values in bold indicate that the difference was significant (*P* < 0.05).

### 3.4 ROC analysis

**Figure 1:**
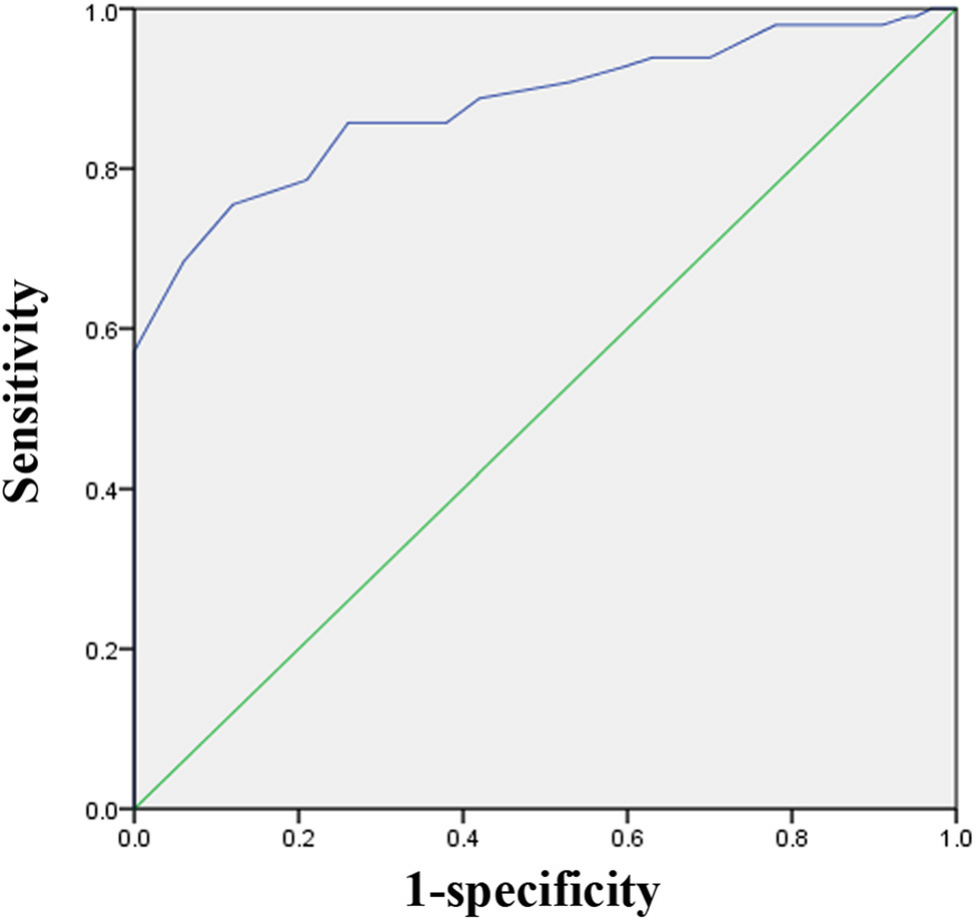
ROC: The sensitivity and specificity of lower uterine myometrial thickness with the incomplete ruptured uterus secondary to previous cesarean.

ROC analysis demonstrated that lower uterine myometrial thickness was linked with the incomplete ruptured uterus secondary to previous cesarean, with an area under the curve of 87.9% (95% CI: 83–92%, *P* ＜ 0.001) as shown in Figure 1. The cut-off values of lower uterine myometrial thickness were determined by selecting the values that produced the highest sensitivity plus specificity combination value. The lower uterine myometrial thickness of 0.64 mm was the cut-off value with the best combination of sensitivity and specificity (75.5 and 88%, respectively) for the incomplete ruptured uterus secondary to previous cesarean.

## 4 Discussion

The study was designed to identify the factors associated with incomplete uterine rupture during delivery of a second pregnancy in a scarred uterus, to compare maternal and infant prognosis with that of an unruptured scarred uterus, to find a way to predict incomplete uterine rupture in scarred uteri. The analysis showed that the likelihood of incomplete uterine rupture secondary to previous cesarean section was associated with the following factors: advanced maternal age, delivery interval ≤36 months, pregnancy with complications, which were consistent with the results of other studies [3,10]. The myometrium, like muscles in other parts of the body, may undergo age-related morphologic changes accompanied by a decrease in tissue elasticity. The results of uterine biopsies suggested an increase in the cholesterol content of muscle cells and an increase in the connective tissue between muscle bundles with age [11]. The risk of uterine rupture increases with shorter interval between cesarean pregnancies, with a sharp decline in the rate of uterine rupture until the tenth month of interval, then a moderate and steady decline until a very moderate decline after the fortieth month [12]. Wound healing in the myometrium is associated with multiple complex cellular interactions, of which the mechanisms of abnormal uterine healing and myometrial rupture remain unclear [13]. Poor healing of the uterine scar after a previous cesarean section may lead to thinning of the anterior uterine wall. Cesarean scar defect (CSD) of the lower uterine segment then occurs and its presence becomes a fatal problem, especially in the case of the next pregnancy.

A study comparing maternal and children outcomes in complete uterine rupture with incomplete uterine rupture suggested a significantly higher need for blood transfusion, more frequent puerperal complications, and higher rates of neonatal asphyxia and transfer to the neonatal unit in women with complete uterine rupture [14]. However, few cases of incomplete uterine rupture secondary to a previous cesarean delivery have been specifically compared with maternal and child outcomes in cases of previous cesarean delivery without uterine rupture. In our study, the number of preterm birth, transfer to the NICU, and placental adhesions were higher, the operation time and the length of time in hospital were longer in the group with incomplete uterine rupture than in the control group.

The absence of peritoneal signs in incomplete uterine rupture may delay the diagnosis, especially if there is little or no abdominal bleeding [13]. This study also found that most incomplete uterine ruptures do not have obvious clinical symptoms. Currently, obstetricians work with ultra-sonographers in clinical practice. This has also become a safe and reliable method for clinicians to predict the risk of uterine rupture, and to combine it with the women’s conscious symptoms for a comprehensive analysis to guide clinicians to terminate the pregnancy at the right time [15]. The definition of uterine incision thinness has varied in many previous studies. Nagy Afifi’s study concluded that total lower uterine segment thickness of <3.65 mm is considered a thin scar, and <2.85 mm is associated with a higher risk of uterine dehiscence [16]. Alalaf et al.’s research showed that a lower uterine segment thickness of 2.3 mm and myometrial thickness of 1.9 mm are associated with a high risk of uterine defects [17]. While a meta-analysis indicated that an lower uterine segment thickness of less than 2 mm likely identifies women at a higher risk of uterine rupture [15]. We usually determine the risk of rupture of the uterine incision based on a combination of the thinness and continuity of the lower myometrium. In our study, results showed that pregnant women with a previous history of lower uterine segment cesarean delivery had an increased risk of incomplete uterine rupture when the thickness of the lower uterine segment myometrium was less than 0.64 mm.

A pathological cardiotocogram (CTG) should lead to particular attention on threatening uterine rupture [18]. A recent work showed that the risk for neonatal acidemia increases very rapidly when pathologic CTG is found [19]. More so bradicardia and long second stages are also substantially associated to neonatal acidemia and to catastrophic events such as uterine rupture [19,20].

CSD, also known as niche, isthmocele, uteroperitoneal fistula, and uterine diverticulum, is a known complication after cesarean delivery [21]. As there are no definitive criteria for diagnosing an isthmocele, several imaging methods can be used to assess the integrity of the uterine wall and thus diagnose an isthmocele [22]. Isthmocele is usually asymptomatic, but its main symptom is abnormal or postmenstrual bleeding, chronic pelvic pain can also occur, and uterine rupture can be one of the complications of this condition [22].

Since this study included elective cesarean section without vaginal trial labor, the patient did not enter labor, there were no data for duration of labor (particularly second stage) in the results and consider assessing this covariate as well as CTG abnormalities. Because the sample size included in this study was limited, it was not a prospective randomized controlled study, and no reproducibility of ultrasound measurements was performed, the reliability of the measurements should be considered.

## 5 Conclusion

In general, according to the current study, for women with a history of at least one cesarean section, there are increased risks of incomplete uterine rupture at age >35 years, delivery interval ≤36 months, pregnancy with complications. For pregnant women at high risk of uterine rupture in late-trimester, it is necessary to combine ultrasound findings of the lower myometrium, maternal conscious symptoms, and the results of fetal heart monitoring to terminate the pregnancy at the appropriate time according to the condition of the mother and child to avoid adverse medical outcomes.
